# Single-cell spatial immune profiling for precision immunotherapy in Lynch syndrome

**DOI:** 10.1016/j.jncc.2024.12.002

**Published:** 2024-12-06

**Authors:** Ramadhani Chambuso, Stephene S Meena

**Affiliations:** 1 Department of Global Health and Population, Harvard T.H. Chan School of Public Health, Boston, United States; 2 Jiangzhong Cancer Research Center, Jiangxi University of Chinese Medicine, Nanchang, China

## Abstract

Lynch syndrome (LS) is the most common hereditary colorectal cancer (CRC) predisposition syndrome, characterized by a high mutational burden and microsatellite instability-high (MSI-H) tumors. Immunology of LS-associated CRC (LS-CRC) is unique, with significant implications for treatment. Despite well-established knowledge of LS immunology, immunotherapy dose and treatment response can vary significantly based on local tumor immunity and specific germline pathogenic variant of LS genes. This variability necessitates tailored surveillance strategies and new personalised immunotherapy approaches for LS patients. LS-CRC often benefits from immunotherapy due to the distinct tumor microenvironment (TME) and the variety of tumor infiltrating lymphocytes (TILs). This perspective discusses a novel approach of analysing spatial TILs at a single-cell level using tumor whole slide images (WSIs) that accounts for the distinct TME of LS-CRC. By emphasizing the necessity of personalized medicine in hereditary cancer syndromes, the future research and clinical practices that enhance patient outcomes through precision oncology is inspired.

## 1 Introduction

Recent advancements in cancer immunotherapy have revolutionized the treatment landscape for colorectal cancer (CRC) in Lynch syndrome (LS) patients.^1^ LS is the most common hereditary CRC predisposition syndrome.^2^ It is an inherited disorder characterized by presence of germline pathogenic variant (GPV) in the four mismatch repair (MMR) genes (*MLH1, MSH2, MSH6, PMS2*) and the *EPCAM* gene. Also, LS tumors are associated with high microsatellite instability (MSI-H) and a pronounced mutational burden.^3^ The unique tumor immunology of high mutational burden and MSI-H tumors and its role in Lynch-associated carcinogenesis has been known for some time.^4^ This unique tumor immunology makes LS-associated CRC (LS-CRC) an ideal candidate for immune checkpoint inhibitors (ICIs) like PD-1/PD-L1 and CTLA-4 inhibitors.^5^ However, the clinical phenotype, cancer severity and inter-patient variability in immunotherapy response may varies by GPV of specific LS genes.^6^, ^7^, ^8^ This in turn has implications on targeted surveillance strategies for LS patients. Even the treatment for some LS-CRC usually change with the addition of immunotherapy for advanced disease and different tumor microenvironment (TME) .^8^^,^^9^ The existing problem is that, traditional biomarkers, such as MSI-H status, mutational load and manual estimation of tumor immune markers provide inconsistent predictive power for immunotherapy response.^10^^,^^11^ Due to the fact that LS-CRC tumors are highly immunogenic, the molecular features render these tumors often lead to an abundant presence of tumor-infiltrating lymphocytes (TILs).^12^ The pivotal role of TILs is in orchestrating anti-tumor immune responses.^13^ In this era, artificial intelligence (AI)-based pathology can characterise immune profile of tumors direct from the tumour histology.^14^^,^^15^ Therefore, to address the above-mentioned existing problem in LS-CRC, we propose a novel integration of leveraging AI to perform detailed immune profiling of these tumors. For the first time this analysis is proposed to focus at a single-cell level spatial TILs as predictors for immunotherapy dose and treatment response. This approach not only refines patient stratification for tailored immunotherapy strategies but also enhances our understanding of the detailed tumour-immune microenvironment in LS-CRC.

## 2 Advancements in single-cell profiling in cancer immunotherapy

Single-cell immune profiling in the TME has revolutionized cancer immunotherapy. This is by providing granular insights into the TME, particularly in highly immunogenic tumors such as LS tumors. Recent advancements in AI have enabled detailed, high-definition (HD) resolution analysis of the TME for individual spatial immune cells.^16^^,^^17^ This uncovers cellular heterogeneity and spatial dynamics of the TILs within the TME with high precision. Single-cell immune profiling of the TME offers transformative potential using AI-driven techniques, such as quantification of spatial TILs in the TME using digital tumor whole-slide imaging (WSI). By elucidating immune cell arrangements, quantity and interactions within the TME, this approach can 1) enhance our understanding of the spatial immune dynamics for each single cell in the TME in relation to immunotherapy outcomes; 2) identify key drivers of immunotherapy resistance, such as tumor-induced immunosuppression in the TME at a single-cell level; 3) enable tailored treatment regimens that account for the unique immune landscapes in the TME of individual LS tumors. Leveraging single-cell spatial immune profiling of the TME in the context of LS immunology, bridges critical knowledge gaps, offering new opportunities to optimize ICI therapies and predict clinical outcomes in LS patients.

## 3 Immune cells in the blood circulation and TME in LS

The TME in LS tumors is characterized by a unique interplay between the high immunogenicity of MMR-deficiency (MMR-D) and the host immune response. LS-CRC exhibit MSI-H status and an elevated tumor mutational burden (TMB), leading to the production of numerous neoantigens in the peripheral blood circulation as the end product of frameshift mutations.^18^^,^^19^ Frameshift mutations are recognized as foreign and immunogenic by the immune system.^20^, ^21^, ^22^ This immunogenic profile fosters a robust infiltration of TILs, particularly CD8^+^ cytotoxic T cells, which are central to anti-tumor immunity. However, the immune TME in LS is highly dynamic, shaped by factors such as immune checkpoint expression (e.g., PD-1/PD-L1), stromal interactions, and tumor-induced immunosuppression, which can modulate the efficacy of immune responses.^12^^,^^13^

Cellular immune responses of T cells and humoral immune responses of B cells are known to be stimulated by exposure to antigens, including pathogens, allergens, and neoantigens. These immune responses help to control tumor growth and spread in LS-CRC.^20^ Monitoring individual patient's immune repertoire in the TME will allow a better understanding of the immune health status of LS-CRC tumors and their response to immunotherapies.

In our two previous publications, we hypothesised on LS variant heterozygotes and persistent high immune responses in both tumor and the peripheral blood.^19^^,^^23^ This is because LS tumors always produce frameshift mutations. These frameshift mutations lead to post-replication defects and the production of neoantigens that cause pre-symptomatic hyper immune responses in individuals with LS. We previously observed that cancers attributable to infections are the rarely reported cancers in LS variant heterozygotes.^23^ This could be due to continuous stimulation of the immune responses in the blood caused by the persistent production of neoantigens.^19^^,^^24^ Our previous hypotheses have the potential to inform future research in precision use of neoantigens in immunotherapy and alternative strategies for vaccine development in LS.

## 4 AI-TILs spatial modelling at a single-cell level

The AI-TILs score represents a cutting-edge approach for quantifying TILs in the tumor microenvironment using histological sections which is derived from sophisticated spatial modelling.^16^ Lymphocytes are meticulously identified and subclassified based on their spatial proximity to epithelial cell nests within hematoxylin and eosin (H&E) stained sections.^16^ Unlike traditional pathology assessments that rely on manual counting or simple area-based measurements, the AI-TILs score leverages machine learning algorithms to analyze the precise spatial distribution and density of lymphocytes relative to cancer cells.^16^ This proximity-based scoring system provides a nuanced measure of immune infiltration. It highlights areas where immune cells are in close contact with tumor cells, which is crucial for understanding immune-tumor interactions at a single cell level. This approach offers a more detailed and reproducible metric of immune infiltration using TILs assessments. It enhances its validity and utility in predicting immunotherapy responses. By integrating this spatially explicit TIL analysis, the AI-TILs score facilitates a deeper understanding of the immune landscape within tumors, aiding in the refinement of immunotherapy strategies and the development of more personalized cancer treatments.

At the single-cell level, the AI-TIL score captures the precise locations of individual lymphocytes and their interactions with surrounding tumor cells and stromal elements.^16^^,^^25^ This level of detail allows for the identification of specific immune cell types and their functional states, enhancing the accuracy of immune infiltration metrics. The single-cell AI-TIL score thus provides a refined approximation of traditional pathology TIL assessments, with significant correlations observed particularly in prognostic cutoffs (e.g., < 30 % vs. ≥ 30 % lymphocyte infiltration) .^16^ High AI-TIL scores suggest that lymphocytes are effectively positioned to recognize and eliminate cancer cells, influenced by the unique ecological and evolutionary dynamics of each tumor.^26^^,^^27^ Utilizing AI-deep learning unsupervised clustering techniques, this approach distinguishes lymphocytes into subsets such as adjacent-tumor lymphocytes, intra-tumor lymphocytes, and distal-tumor lymphocytes. Finally, the total AI-TILs score is computed by taking the ratio of adjacent-tumor lymphocytes to the total fibroblast count, reflecting the density and spatial arrangement of immune cells in relation to stromal components.^16^

In parallel, the cancer-immune cell colocalization metric, quantified using the Morisita-Horn index, the spatial overlap will be evaluated between identified cancer and immune cells at single-cell resolution. This measure assesses the degree to which individual immune cells co-localize with cancer cells, providing insights into the structural and functional organization of immune infiltration. High colocalization values indicate frequent interactions between immune and cancer cells, essential for effective immune surveillance and synapse formation. The single-cell approach allows for the detailed mapping of immune cell distribution, capturing variations in cell density, type, and functional engagement within the TME.

The advantages of spatial single-cell analysis of TILs including: 1) offers granular details with high-resolution data on the spatial positioning and interactions of immune cells with tumor cells^28^; 2) accurately captures the cellular heterogeneity within the TME, identifying all diverse immune subsets and their specific roles.^29^^,^^30^ 3) enables the assessment of immune cell states and their functional insights, such as activation, exhaustion, or regulatory roles, which are critical for understanding immune dynamics and immunotherapy response.^31^ 4) enhances precise predictive accuracy for immunotherapy outcomes by correlating detailed spatial immune infiltration within the TME and functional data for personalised immunotherapy with clinical responses to minimise adverse effects.^32^^,^^33^

**Fig. 1:**
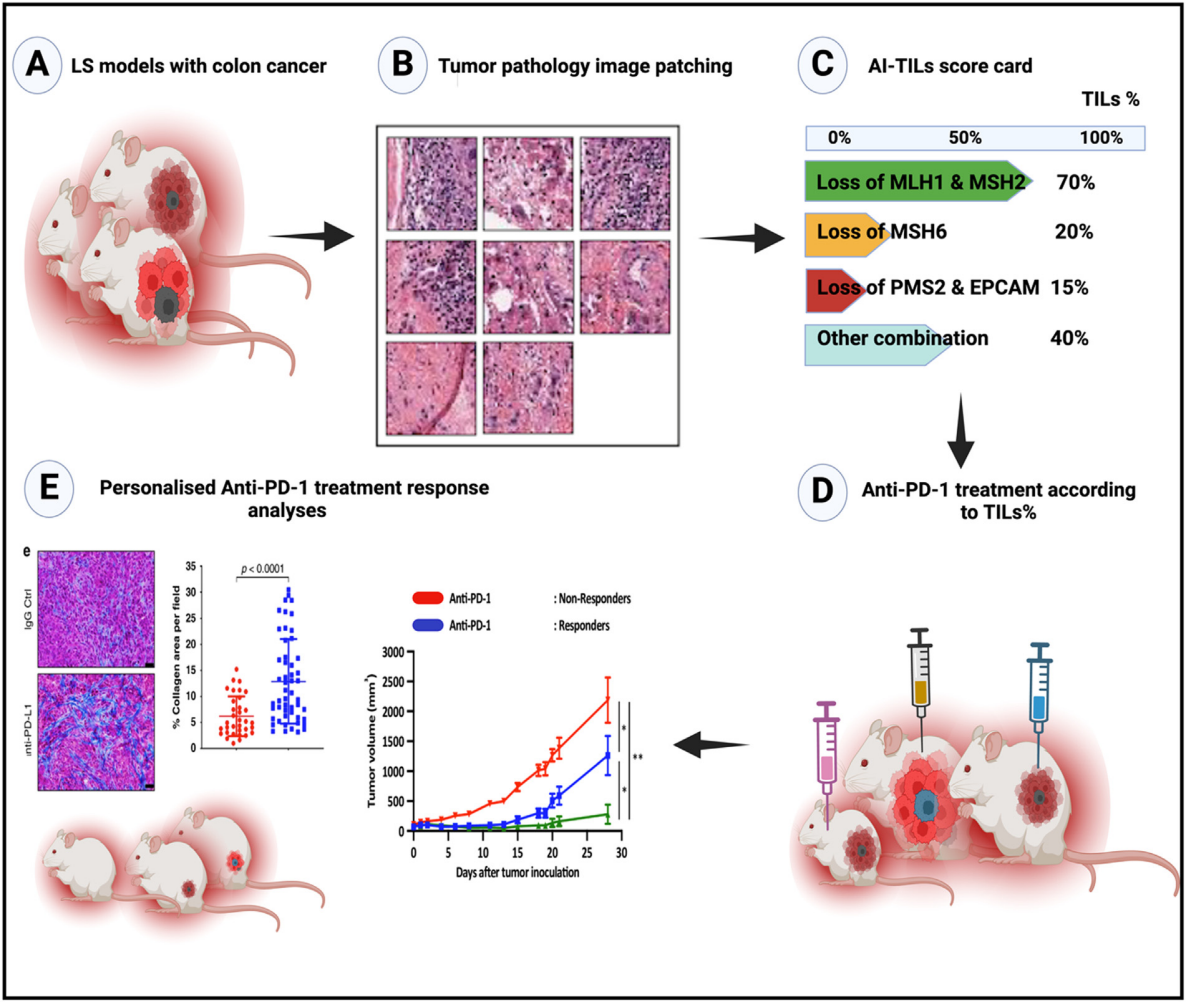
AI-Enhanced TILs profiling for personalized immunotherapy in LS-CRC. (A) Genetically engineered mouse models mimicking LS with induced colon cancer to study the tumor-immune microenvironment and therapeutic responses. (B) Tumor pathology image patching to show the segmentation of histological slides from LS tumors into high-resolution patches in pixels, displaying diverse histological features and immune cell infiltration patterns for detailed analysis of TILs at a single-cell level. (C) AI-TILs score card analysis resents an AI-generated TILs scorecard categorizing LS tumors by TILs percentages linked to specific LS genetic mutations, with high TIL presence in tumors with MLH1 & MSH2 loss (70 %) and varying levels for other mutations. (D) Anti-PD-1 treatment according to TILs% to illustrate the use of AI-derived TILs percentages at a single cell level to guide treatment. (E) Personalized anti-PD-1 treatment response analyses to show different correlations between TIL scores and anti-PD-1 treatment response, with histological images and graphs depicting significant tumor volume reductions in overall TIL-high tumors treated with anti-PD-1 therapies, underscoring the potential of AI-driven TILs profiling for personalized immunotherapy in LS-CRC. AI-TILs, artificial intelligence- tumor-infiltrating lymphocytes; LS-CRC, Lynch syndrome-associated colorectal cancer.

A demonstration of our conceptual framework employs LS mouse models with colon cancer to evaluate the immune landscape and response to anti-PD-1 immunotherapy (Fig. 1). Using an established AI algorithms to perform image patching of tumor pathology H&E slides to quantify TILs, revealing distinct patterns of immune infiltration. Creation of TILs-AI scorecard which classifies tumors into categories with varying levels of TILs presence, highlighting a substantial proportion of LS tumors with high TIL scores.^34^ This stratification is instrumental in predicting treatment response, as evidenced by the differential outcomes observed in anti-PD-1 treated cohorts.

## 5 Unresolved novel questions and proposed research directions

Despite the progress in immunotherapy and AI-driven TILs profiling, several critical questions remain unanswered in the field. Addressing this through novel research questions could significantly enhance our understanding and treatment outcomes of these patients.

a) **Question 1**: How does the spatial heterogeneity of TILs within LS tumors influence immunotherapy outcomes?^16^^,^^35^

- **Rationale**: While overall TIL density is a known predictor, the impact of their spatial distribution within different tumor regions on therapy response is not well understood.^36^^,^^37^

- **Specific objective**: Develop advanced AI models to analyze the spatial organization of TILs relative to tumor nests and stromal regions, correlating these patterns with clinical outcomes.^38^^,^^39^

- **Justification**: Enhancing TILs spatial analysis by developing sophisticated AI algorithms that can not only quantify TILs density but also analyze their spatial arrangement within tumors, providing a more comprehensive predictive tool for therapy response and personalised dose.^40^^,^^41^

b) **Question 2**: What is the extent of the variability of the mechanisms driving the differential immune infiltration patterns observed in different LS-CRC cases according to the specific MMR deficient gene?^42^^,^^43^

- **Rationale**: The molecular and cellular mechanisms underlying distinct TILs populations at a single-cell level in different MMR deficient genes in LS patients are not fully elucidated.^43^

- **Specific objective**: Conduct comparative studies using single-cell RNA sequencing and spatial transcriptomics to dissect the immune cell dynamics and interactions according to specific MMR deficiency genes in LS patients.^44^

- **Justification**: Comparative immune profiling can be used to integrate multi- omics approaches, such as single-cell sequencing and spatial transcriptomics, to compare immune landscapes between LS patients according to the specific germline MMR deficient gene.^44^^,^^45^

c) **Question 3**: Can AI-driven TIL percentage spatial profiling predict personalised dose and resistance to ICIs?^46^

- **Rationale**: Predictive markers for personalised immunotherapy dose, adverse effects and resistance are inadequately characterized.^47^

- **Specific objective**: Utilize AI to identify TIL profiles associated with the response for resistance and integrate this data with genomic and proteomic analyses to uncover resistance mechanisms, adverse effects, and potential personalised dosage.^48^

- **Justification**: Identifying resistance mechanisms by implement AI to correlate

TIL profiles with resistance patterns and use this data to explore combination therapies that might overcome observed resistance and suggest personalised efficient dose.^49^^,^^50^

d) **Question 4**: How do alterations in the TME induced by ICIs affect subsequent tumor immune responses in LS-CRC?^51^

- **Rationale**: The dynamic changes in the TME following ICI treatment and their impact on subsequent immune activity have not well-defined.^52^^,^^53^

- **Specific objective**: Employ longitudinal studies using AI to monitor TME alterations over time, combined with functional assays to assess the immune cell activity post-immunotherapy treatment.^54^

- **Justification**: Monitoring TME dynamics to utilize real-time imaging and AI analysis to track changes in the TME post-ICIs treatment, offering insights into immune cell behaviour and potential reprogramming strategies.^55^^,^^56^ This can be achieved without being invasive using several advanced techniques that can be employed after completion of immunotherapy such as: 1) Multiparametric magnetic resonance image (MRI) which utilizes various MRI techniques (e.g., diffusion-weighted imaging, dynamic contrast-enhanced MRI) to visualize changes in the TME, such as immune cell infiltration and tumor vascularity.^57^ 2) Positron emission tomography (PET) and computed tomography (CT) with immune cell-specific tracers that use radiolabeled tracers targeting specific immune cells (e.g., CD8^+^ *T* cells) to assess their presence and activity in the tumour.^57^^,^^58^ 3) Ultrasound with contrast agents injected into the tumor region e.g. microbubble contrast agents that can highlight vascular changes and immune cell trafficking within the tumor.^59^^,^^60^

e) **Question 5**: What role do non-TIL immune components, such as macrophages and dendritic cells, play in modulating immunotherapy responses in LS-CRC?^61^^,^^62^

- **Rationale**: The contribution of non-TIL immune cells to the immunotherapy response in LS-CRC remains largely unexplored.^63^

- **Specific objective**: Expand AI-based analysis to include the identification and profiling of other immune cell types within the TME, evaluating their interactions with TILs and their impact on therapy outcomes.^64^

- **Justification**: Broadening immune profiling by extending AI-driven analysis to encompass a wider range of immune cells. This will provide a holistic view of the TME and identifying novel targets for enhancing immunotherapy efficacy.^65^

## 6 Constraints of the proposed AI approach

This approach shows significant potential; however, it is not without its limitations. The AI-driven spatial immune profiling technique at the single-cell level for personalized immunotherapy is deemed more effective for highly immunogenic tumors such as LS-CRC, owing to the abondance presence of TILs. However, the applicability of this technique is limited when it comes to non-LS tumors or tumors with poor immune infiltration such as tumors with chronic hypoxia or glucose depletion in the TME. Another notable drawback is the substantial operational costs associated with the utilization of sophisticated techniques and technologies, such as digital high-definition (HD) scanners for digital pathology laboratories with built-in AI systems to quantify spatial TILs in the TMEs automatically from the WSIs. Challenges in the involvement of multiparametric MRI and PET that employ immune cell-specific radiolabelled tracers for the localization and functional characterization of various immune cells within the tumor.^57^^,^^66^ This approach is restricted to evaluating immunotherapy responses in tumors that has already been entirely resolved, lacking malignant tissue encompassing tumor-immune microenvironment necessary for spatial immune profiling. Lack of knowledge about active specific functional or immune signalling pathways can significantly impact the prediction of treatment response in immunotherapy. While the presence of abundant spatial TILs is generally associated with a favourable prognosis and immunotherapy responsiveness, their functional state and the activity of key signalling pathways are critical determinants of their efficacy in mediating anti-tumor immunity. For example TILs may be abundant but functionally exhausted due to chronic antigen exposure, characterized by upregulation of inhibitory receptors (e.g., PD-1, CTLA-4) and reduced cytokine production. This would render the immune cells less effective against cancer cells despite their abondance physical presence.

## 7 Conclusions

This article describes a novel integration of AI-enhanced spatial TILs analysis at a single-cell level as a standard practice for immunotherapy of LS-CRC tumors. This may pave the way for more tailored and transformative effective immunotherapeutic regimens. The present approach represents a significant advancement in precision oncology in immunotherapy. By providing a comprehensive and objective analysis of TILs detail using AI, the described approach addresses the variability in effective dose and immunotherapy responses against the current approach of “same size fits all”, and paves the way for more personalised and effective therapeutic interventions to reduce adverse effects. Comprehensive profiling, including spatial and functional characterization of TILs, their signalling pathway activity, and interaction with the TME, is essential for accurate treatment predictions and tailoring immunotherapy strategies. This integration can identify actionable targets to enhance TIL functionality and improve therapeutic outcomes. We underscore the need for continued exploration of AI applications in oncology to fully realize the benefits of precision medicine in treating genetically predisposed immunogenic cancers like LS-CRC. Addressing unresolved questions through targeted research will enhance our understanding of immune dynamics at TME and improve the predictive accuracy for immunotherapy, ultimately leading to more personalized and effective cancer treatment.

## Declaration of competing interest

The authors declare that they have no known competing financial interests or personal relationships that could have appeared to influence the work reported in this paper.
